# Ultrasound Evaluation of Fontan-Associated Liver Disease: A State-of-the-Art Review

**DOI:** 10.3390/diagnostics15243171

**Published:** 2025-12-12

**Authors:** Federica Di Natale, Andrea Boccatonda, Marco Musmeci, Alice Brighenti, Luciano Potena, Christoph Frank Dietrich, Carla Serra

**Affiliations:** 1 Diagnostic and Therapeutic Interventional Ultrasound Unit, IRCCS Azienda Ospedaliero-Universitaria di Bologna, Policlinico Sant’Orsola-Malpighi, via Massarenti N 9, 40138 Bologna, Italy; 2 Heart Failure and Transplant Unit, IRCCS Azienda Ospedaliero-Universitaria di Bologna, 40138 Bologna, Italy; 3 Department Allgemeine Innere Medizin (DAIM), Kliniken Hirslanden Beau Site, Salem und Permanence, 3013 Bern, Switzerland

**Keywords:** Fontan, liver, FALD, heart, hepatocellular carcinoma

## Abstract

**Background**: Fontan-associated liver disease (FALD) is a progressive condition resulting from chronic hepatic venous congestion following the Fontan procedure for univentricular heart defects. As survival improves in these patients, recognition and management of FALD have become increasingly important. **Objective**: To describe the pathophysiological mechanisms, imaging findings, and diagnostic approach to FALD, with a focus on the role of ultrasonography, including contrast-enhanced ultrasound (CEUS). **Methods**: This narrative review explores the evolution of FALD through a multidisciplinary lens, integrating cardiovascular and hepatic imaging data. Particular attention is paid to Doppler ultrasound and CEUS, both in early parenchymal changes and in the differential diagnosis of potential complications such as hepatic nodules. **Results**: FALD is characterized by progressive fibrosis due to long-standing passive congestion, resulting in a wide spectrum of imaging findings. B-mode ultrasound reveals hepatomegaly, heterogeneous parenchyma, and gallbladder wall thickening. Doppler studies show altered hepatic venous flow patterns, while CEUS provides dynamic vascular evaluation, highlighting areas of altered perfusion. In advanced stages, hypo-vascular areas in the late phase may simulate malignant lesions, emphasizing the need for careful interpretation. The role of liver biopsy, though limited by invasiveness, remains crucial in selected cases. Surveillance strategies are not standardized but require close multidisciplinary follow-up. **Conclusions**: FALD presents complex diagnostic challenges requiring integrated imaging and clinical assessment. CEUS emerges as a valuable, non-invasive tool in characterizing hepatic congestion and guiding management. Increased awareness and standardized protocols are essential for early detection and tailored care in this growing patient population.

## 1. Introduction

**Figure 1:**
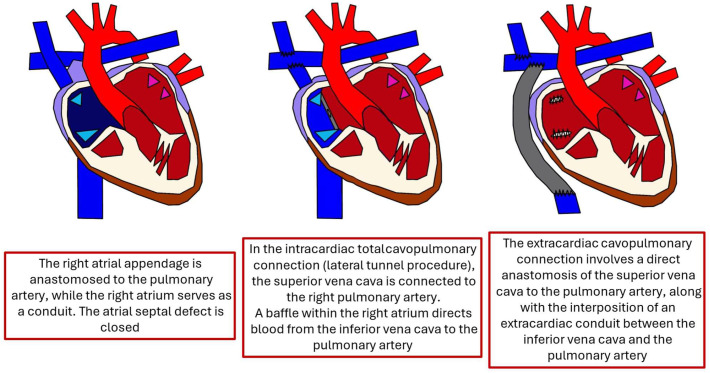
Fontan-type surgical approaches.

The Fontan procedure is a surgical intervention used to treat congenital heart defects resulting in a single functional ventricle, such as hypoplastic left heart syndrome or tricuspid atresia. This procedure diverts systemic venous return directly to the pulmonary arteries, bypassing the heart. The initial version of the Fontan procedure, known as the atrio–pulmonary connection or classical Fontan, was originally developed for patients with tricuspid atresia. This approach involved closing the atrial septal defect and directly linking the right atrium to the right pulmonary artery. Over time, several modifications were introduced (Figure 1), including the revised atrio–pulmonary technique by Kreutzer and the right atrial-to-pulmonary artery connection described by Björk et al. [1,2]. A significant evolution came in 1988 with the introduction of the total cavo-pulmonary connection, or lateral tunnel, by De Leval. In this method, the superior vena cava is connected to the right pulmonary artery (classical Glenn shunt), while the inferior vena cava is routed to the right atrium through an intracardiac tunnel. The most recent development is the extracardiac cavo-pulmonary connection, which involves a direct anastomosis between the superior vena cava and the right pulmonary artery. A vascular conduit is placed externally to link the inferior vena cava to the right pulmonary artery. This technique offers advantages such as avoiding myocardial ischemia, reducing the number of suture lines, and eliminating the use of foreign material within the right atrium.

The Fontan circulation, characterized by the absence of a sub-pulmonary ventricle, fundamentally alters cardiovascular dynamics. In this configuration, systemic venous blood is directed passively into the pulmonary arteries, relying on elevated central venous pressure (CVP) to drive pulmonary perfusion. This passive flow results in a reduced preload to the systemic ventricle, limiting stroke volume and cardiac output. Concurrently, the systemic ventricle faces increased afterload, as it must overcome both systemic and pulmonary vascular resistance without the assistance of a sub-pulmonary pump. Three main mechanisms facilitate the passage of blood from the systemic venous system to the left atrium in the Fontan circulation [3]:

- The suction effect generated by left atrial relaxation during diastole.
- The action of the respiratory and muscular pumps, driven by the negative intrathoracic pressure during inspiration, which enhance inferior vena cava return and promotes pulmonary vessel recruitment.
- The maintenance of low pulmonary vascular resistance, provided there is no mechanical obstruction within the Fontan conduit, the main pulmonary arteries, or the pulmonary veins.

These hemodynamic changes can lead to ventricular dysfunction over time, manifesting as decreased ejection fraction and diastolic impairment. Additionally, the elevated CVP inherent in the Fontan circulation contributes to complications such as hepatic congestion, protein-losing enteropathy, and decreased exercise tolerance. Understanding these alterations is crucial for optimizing long-term management and improving outcomes in patients with Fontan physiology.

These hemodynamic challenges predispose patients to long-term complications, including liver and kidney diseases. The Fontan physiology is characterized by chronically elevated CVP and reduced cardiac output, significantly altering normal organ perfusion and function [3]. These changes can progressively impair the liver and kidneys, organs highly sensitive to hemodynamic stress [3].

Fontan circulation can fail due to systolic or diastolic ventricular dysfunction, atrioventricular valve disease, elevated pulmonary vascular resistance, recurrent arrhythmias, lymphatic insufficiency, or end-organ complications [4]. Accordingly, symptoms may be related to heart failure and hypoxemia or end-organ complications, resulting in different clinical and hemodynamic Fontan failure phenotypes [4] (Table 1).

1. Systolic Failure Phenotype (SFP): The single ventricle’s pumping ability deteriorates, leading to a reduced ejection fraction. Clinically, patients may experience fatigue, ascites and exercise intolerance. Hemodynamically, this group is characterized by a low cardiac index, often less than 2.2 L/min/m^2^, and variable Fontan pressures. These patients are typically the most ill and often require early evaluation for transplant due to poor outcomes with medical therapy alone.
2. Preserved Systolic Function with Elevated Pressure (PFP): In this phenotype, the ventricle itself is functioning normally in terms of contractility, but the Fontan circuit develops resistance. Elevated pressures within the Fontan pathway—often due to increased pulmonary vascular resistance, conduit stenosis, or lack of compliance—lead to systemic venous congestion. Patients may present with peripheral edema, liver congestion, and ascites. Despite a preserved cardiac output, end-organ dysfunction progresses, and clinical management often focuses on relieving congestion or improving Fontan flow dynamics.
3. Lymphatic Failure Phenotype (LFP): Some patients with Fontan circulation develop complications related to abnormal lymphatic flow, even in the absence of severe hemodynamic derangements. These patients may present with protein-losing enteropathy, plastic bronchitis, or chylous effusions. This phenotype is increasingly recognized because of chronic central venous hypertension impairing lymphatic drainage, especially from the gut and lungs. Management often requires targeted lymphatic interventions, such as lymphangiography-guided embolization or specialized dietary and pharmacologic therapies.
4. Normal Hemodynamics Phenotype (NHP): A subset of patients may exhibit clinical symptoms of Fontan failure despite having normal cardiac function and pressures. These cases can be particularly challenging to diagnose and manage, as standard hemodynamic evaluations appear normal. The underlying mechanisms are not fully understood but may involve diastolic dysfunction, subtle microvascular issues, or autonomic imbalance. Despite “normal” measurements, these patients may have reduced exercise tolerance or quality of life and require a multidisciplinary approach to evaluation.

Fontan-associated liver disease (FALD) encompasses a broad spectrum of structural, functional, and clinical liver alterations secondary to Fontan hemodynamic changes [3,5] (Table 2). These changes are due to the pathological hemodynamic state that exists in any type of Fontan circulation from the first day of the surgical procedure, meaning that FALD can also occur in patients without Fontan circulatory failure [3,4]. Liver damage can start even before the Fontan procedure and is present in patients without Fontan circulatory failure [3,4] (Table 3). This review aims to provide an updated overview of FALD and to summarize current knowledge on the role of imaging modalities in the evaluation of liver disease in this population, with a particular focus on ultrasound-based techniques.

## 2. FALD Epidemiology

In 2020, individuals with a history of Fontan-type surgery were estimated to number 66 per million, with forecasts indicating an increase to 79 per million by 2030 [6,7]. That year, the distribution consisted of 55% adults, 17% adolescents, and 28% children. By 2030, these proportions are expected to adjust to 64%, 13%, and 23%, respectively [6]. During the COVID-19 pandemic, gaps in cardiology follow-up significantly increased among patients with Fontan circulation [4,8,9,10]. The precise incidence of FALD remains elusive and likely underreported, reflecting the current absence of a uniform, consensus-based definition [6]. However, the actual prevalence of some of these conditions may be higher due to subclinical disease presentation and the lack of a universal definition. Hispanic ethnicity was associated with increased all-cause mortality [6]. Furthermore, the prevalence of FALD is underrecognized, but there is evidence that its incidence increases with age [4]. Despite its association with late mortality, liver transplant outcomes are excellent [4]. This supports the need for structured, FALD-focused hepatic surveillance in Fontan patients.

## 3. Pathophysiology

FALD develops through a complex combination of hemodynamic, structural, and molecular mechanisms (Table 4). Multiple abnormalities affecting hepatic vascular inflow and outflow have been associated with the onset of liver injury in patients with Fontan disease [7]. In the Fontan population, portal hypertension is predominantly post-sinusoidal, reflecting the passive transmission of chronically elevated central venous pressure to the hepatic sinusoids rather than increased intrahepatic resistance from fibrosis [11]. As a consequence, findings such as splenomegaly, thrombocytopenia, or venous dilation may fluctuate and often partially improve after optimization of cardiac preload and afterload, augmentation of cardiac output, or relief of obstruction within the Fontan pathway [11,12]. Moreover, splenomegaly and thrombocytopenia are common to other chronic liver diseases [13]. This hemodynamic form of PHTN differs fundamentally from that driven by intrinsic hepatic architectural distortion, and careful distinction between congestion-related and fibrosis-related portal hypertension is essential [11]. This differentiation carries major implications for clinical decision-making, particularly when considering heart versus combined heart–liver transplantation and when evaluating the reversibility of end-organ dysfunction

Systemic venous hypertension is transmitted to the hepatic sinusoids through the hepatic veins, leading to sinusoidal dilatation, hyperfiltration, and perisinusoidal edema [14,15]. The resulting increase in sinusoidal shear stress promotes endothelial capillarization, lowers intrahepatic levels of vasodilators, and activates mechanosensitive signaling pathways in hepatic stellate cells, ultimately contributing to hepatic fibrosis [16,17,18]. These alterations impair the delivery of oxygen and metabolic substrates, predisposing to centrilobular hepatocyte loss and tissue atrophy [19].

Increased CVP can impair the hepatic arterial buffer response, diminishing its compensatory capacity [5,20]. As a result, the liver becomes more susceptible to hypoxic injury during acute events (such as cardiac surgery or episodes of cardiopulmonary collapse) [21,22]. Over time, persistently reduced cardiac output together with the formation of abnormal intrapulmonary shunts would further contribute to sustained hepatic hypoxia [21,22].

The impaired functional and anatomical characteristics of the Fontan circulation and the acquired thrombophilic state (high levels of thrombin–antithrombin complex, low serum levels of C and S proteins, thrombomodulin, antithrombin III and alpha-2-antiplasmin) may increase intrahepatic microthrombosis [23,24,25].

Enhanced hepatic lymphangiogenesis together with lymphatic stasis contributes to marked dilation of the hepatic sinusoids, the space of Disse, and the channels traversing the limiting plate, changes that may ultimately favor collagen deposition [26,27]. A persistent, low-grade inflammatory milieu may further drive fibrogenic processes in affected organs. In addition, increased intestinal permeability caused by venous and lymphatic congestion and by chronic ischemia is thought to play a key role in sustaining inflammation. Other contributing factors include the higher prevalence of hepatitis B and C infections and the potential for drug-induced hepatotoxicity, such as that associated with amiodarone use [28].

## 4. Clinical Presentation

FALD most often develops sub-clinically during the early phases. Clinically, some patients can report pain or discomfort in the upper right quadrant of the abdomen due to the distension of the capsule of Glisson [7,29]. The liver edge is easily palpable, firm, smooth, and tender in these cases. Hepatojugular reflux may also be easily identified after applying compression over the liver [30]. Mild jaundice is often present, while severe jaundice is a rarer occurrence (such as at the end of an episode of hypoxic hepatitis) [7,30]. Even when FALD progresses to advanced stages and cirrhosis is present—though still compensated—patients may remain asymptomatic or display only vague manifestations such as anorexia, fatigue, or unintended weight loss [30]. When liver disease becomes decompensated, features such as ascites, jaundice, variceal hemorrhage, and hepatic encephalopathy may emerge. These findings indicate the development of portal hypertension (PHTN), a condition linked to poorer clinical outcomes in individuals with Fontan circulation [7,29]. Episodes of variceal bleeding have been documented after the Fontan procedure, with reported rates varying widely from 9.3% to 38% [29]. Among pediatric patients, varices occur far less frequently—around 9%—supporting the notion that they tend to arise as a later complication [31]. Given the high mortality risk associated with this complication (15–20%), upper gastrointestinal endoscopy is commonly recommended for adult patients with cirrhosis or signs of PHTN [32].

## 5. Diagnosis

The diagnosis of FALD requires a comprehensive and integrated approach, as no single test can definitively identify or stage the condition. Evaluation typically begins with a thorough clinical assessment, including history and physical examination [33]. Clinicians look for signs of chronic liver disease such as hepatomegaly, ascites, jaundice, spider angiomas, and splenomegaly [33]. Symptoms may be subtle in early stages and include fatigue, abdominal discomfort, or evidence of portal hypertension, like gastrointestinal bleeding [33]. Laboratory testing plays a central role in the diagnostic process (Table 5). Liver enzymes such as ALT and AST may be normal or only mildly elevated, while markers of cholestasis (such as alkaline phosphatase and GGT) and bilirubin levels can provide additional insight [34]. Tests of synthetic liver function, including serum albumin and the international normalized ratio (INR), are useful for assessing disease severity [34]. A complete blood count may reveal thrombocytopenia, often a sign of portal hypertension, and serologic testing helps to exclude other causes of liver disease, such as viral hepatitis or autoimmune hepatitis [34]. Common fibrosis scores like APRI and FIB-4 are frequently used in other chronic liver diseases but have limited accuracy in FALD due to the unique hemodynamics of the Fontan circulation [34].

Imaging is a cornerstone of noninvasive evaluation. Abdominal ultrasound is often the first-line modality, allowing for assessment of liver size, texture, nodularity, and signs of portal hypertension such as splenomegaly or ascites [35,36]. Doppler ultrasound adds valuable information by evaluating hepatic and portal venous waveforms, which can reflect changes in venous congestion or progression toward cirrhosis [36,37]. Liver elastography, through either transient elastography or shear wave techniques, provides an estimate of liver stiffness [37]. However, stiffness values may be confounded by hepatic congestion and must be interpreted cautiously. Cross-sectional imaging with contrast-enhanced computed tomography (CT) or magnetic resonance (MR) offers detailed structural information and helps detect liver nodules, vascular changes, or collateral circulation [38]. MR with hepatobiliary contrast agents and MR elastography can offer superior tissue characterization, though their availability may be limited in some centers [38].

Endoscopic evaluation is recommended in patients at risk of portal hypertension [39]. Upper gastrointestinal endoscopy allows for direct visualization of esophageal and gastric varices, which may be asymptomatic until bleeding occurs [39]. In select cases, invasive testing is necessary [40]. Liver biopsy remains the gold standard for assessing fibrosis and confirming cirrhosis [40,41]. Histological findings in FALD typically include sinusoidal dilation, perivenular and perisinusoidal fibrosis, and in advanced stages, nodular regenerative hyperplasia [42]. However, in the case of FALD, this procedure is more challenging due to the potential for sampling errors caused by the heterogeneous nature and irregular distribution of fibrosis [42]. Additionally, as an invasive technique, liver biopsy poses a heightened bleeding risk due to anticoagulant therapy and high CVP, limiting its feasibility for repeated evaluation over time. In children, the procedure typically necessitates sedation or even general anesthesia, adding further procedural complexity. For these reasons, noninvasive approaches to diagnosis and staging represent a compelling alternative [43]. While hepatic venous pressure gradient (HVPG) is a standard tool in other liver diseases, it is less useful in the Fontan population due to altered vascular anatomy and hemodynamics. Conversely, cardiac catheterization remains an important tool to assess Fontan pressures and exclude hemodynamic contributors to liver congestion. Finally, in patients with advanced disease or cirrhosis, surveillance for hepatocellular carcinoma is crucial [44,45]. This typically involves regular imaging—such as ultrasound or MRI—every 6 to 12 months, sometimes paired with serum alpha-fetoprotein (AFP), although AFP alone has limited sensitivity and specificity [44,45].

## 6. Imaging

Imaging plays a valuable role in the diagnosis and surveillance of FALD [3]. Common imaging findings in patients with FALD include a heterogeneous liver texture. Initially, hepatomegaly is likely to develop because of venous congestion. With the development of advanced cirrhosis, atrophy of the right lobe with hypertrophy of the caudate lobe are seen, together with dilation of hepatic veins, IVC, and signs of PHTN, such as ascites, splenomegaly, and varices [3,7]. The specific atrophy of the right hepatic lobe in advanced cirrhosis can be explained by a combination of decreased portal and arterial inflow, intrahepatic anatomical changes and compensatory hypertrophy of the left lobe.

In advanced cirrhosis, there is significant disruption of hepatic blood flow, particularly a reduction in portal vein inflow to the right lobe. With the development of portal hypertension and portosystemic shunting, the right lobe becomes more susceptible to hypoperfusion, leading to chronic ischemia and atrophy. Structural changes in the liver (fibrosis, regenerative nodules, intrahepatic shunts) can compress portal branches supplying the right lobe. In some cases, a selective thrombosis of the right portal vein branch can be observed. As chronic liver disease progresses, the left lobe may become hypertrophic and more metabolically active, compensating for the reduced function of the right lobe. This is part of the compensatory atrophy–hypertrophy complex, where the right lobes shrink, and the left lobe enlarges.

Individuals showing imaging features compatible with PHTN face a markedly elevated risk—up to nine times higher—of developing hepatocellular carcinoma, requiring liver transplantation, or experiencing mortality. Although hepatomegaly is readily identifiable on imaging, hepatic fibrosis typically remains occult until it reaches more advanced stages. Characteristic structural changes of cirrhosis—including segmental atrophy, caudate lobe enlargement, and accentuated nodularity—may be concealed in the early phases because congestion-induced hepatic enlargement can mask fibrotic volume loss. Fibrosis in these patients is often unevenly distributed and may present as numerous peripheral nodules of differing dimensions, detectable in both early and advanced disease [3,7]. With ongoing disease progression, cirrhosis may lead to loss of right-lobe volume and increasing surface nodularity [3,7]. In most cases, fibrosis or cirrhosis exhibits an irregular, discontinuous pattern rather than a uniform distribution [3,7]. Additionally, venovenous shunts, a dilated inferior vena cava, and engorged hepatic veins are frequently observed due to elevated CVP [3,7]. The combination of elevated CVP and low cardiac output leads to hepatic ischemia.

## 7. B-Mode & Color-Doppler Findings

**Figure 2:**
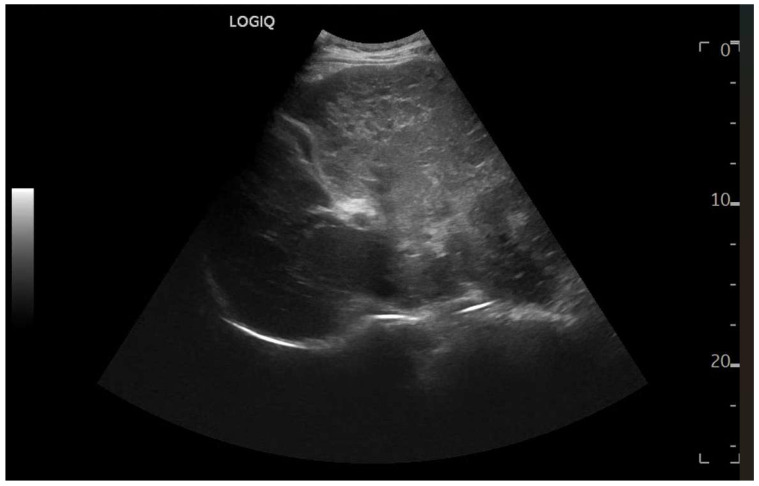
Detail with convex probe in oblique subcostal scan: note the rounded margins, the heterogeneous echotexture, particularly in the left segments, and a marked hypotrophy of the right lobe.

**Figure 3:**
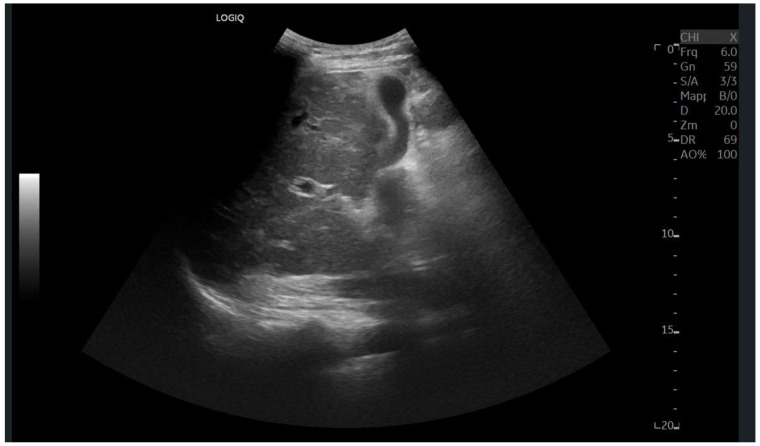
Subcostal scan with convex probe showing symmetrical thickening of the gallbladder walls, consistent with congestion.

**Figure 4:**
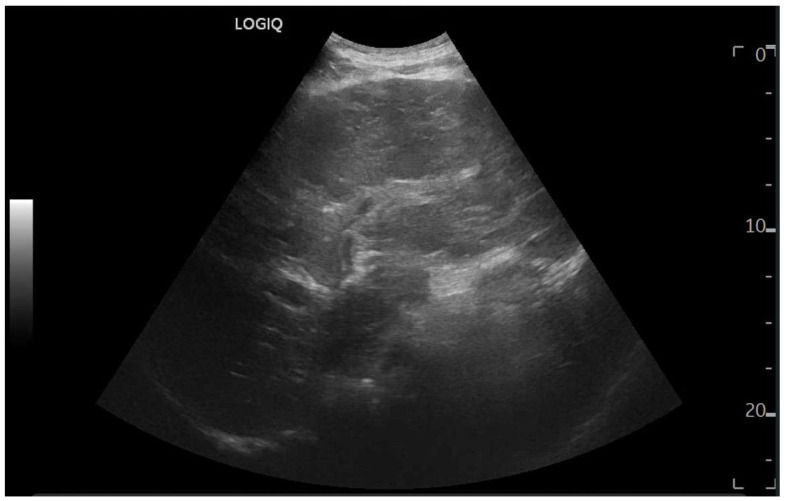
Oblique subcostal scan with convex probe showing diffuse periportal edema.

**Figure 5:**
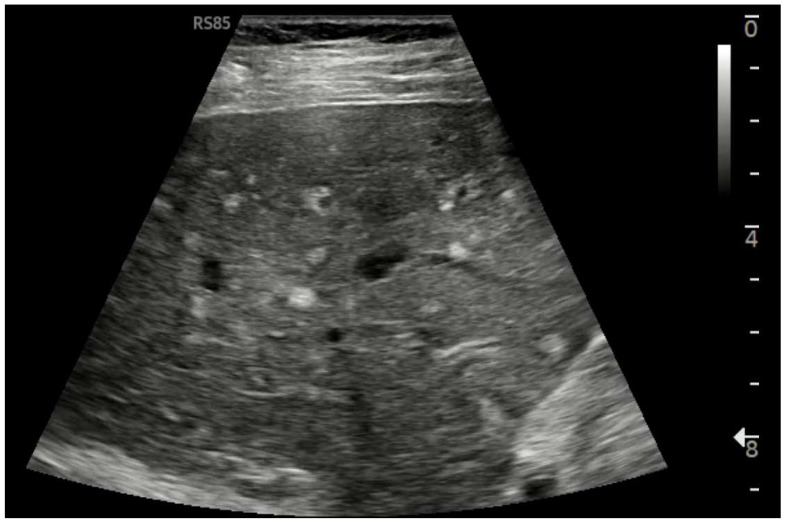
Linear Probe (9 MHz) with trapezoidal scan. Diffusely dense and heterogeneous echostructure due to the presence of multiple and diffuse hyperechoic areas, suggestive of peri-portal fibrosis.

**Figure 6:**
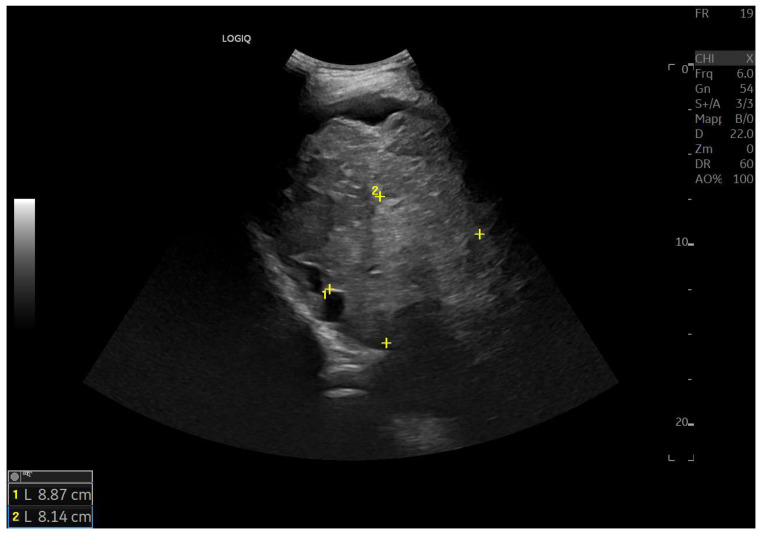
Diffuse echostructural change of the left lobe with a heterogeneous hyperechoic area measuring 89 × 81 mm.

**Figure 7:**
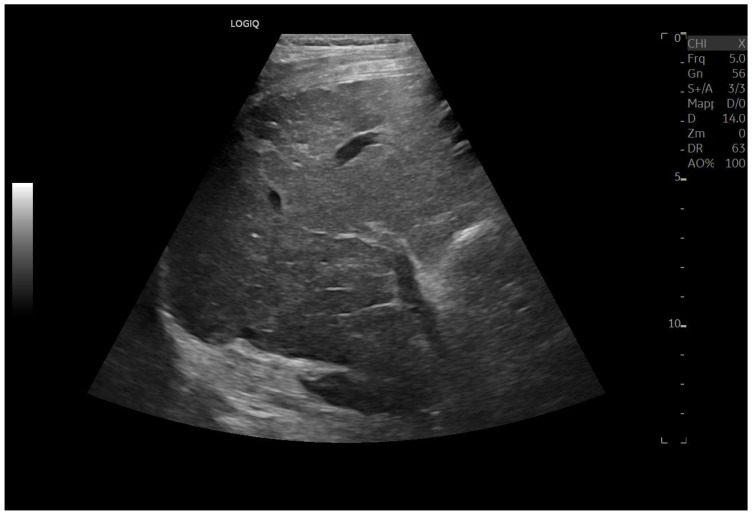
Hypotrophy of the right lobe. Nodular profiles. Markedly heterogeneous echostructure, with increased fibrous component and fibrous trabeculae delimiting pseudo-nodular areas, within the context of a vascular-type cirrhosis.

**Figure 8:**
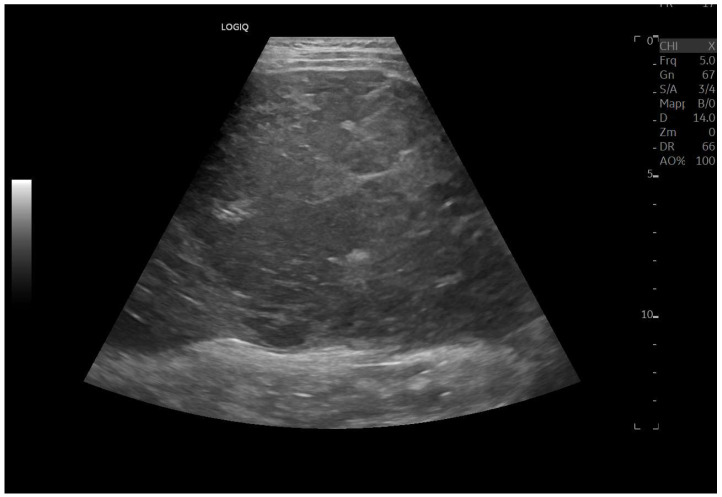
Marked hypertrophy of the left lobe, which partially occupies the left hypochondrium.

Ultrasound imaging is capable of identifying heterogeneous echotexture and hepatomegaly, which are early indicators of congestive hepatopathy [46]. Several studies have shown correlations between the extent of sonographic abnormalities and the severity of hepatic fibrosis or cirrhosis [36,47,48]. The most common ultrasound findings encompass a nodular hepatic surface, right-lobe volume reduction, smooth-to-rounded contour changes, and irregular outer profiles [49,50,51] (Figure 2). The echotexture appears granular and markedly heterogeneous, with hyperechogenic nodules of varying sizes [49,50,51] (Figure 3, Figure 4, Figure 5, Figure 6, Figure 7 and Figure 8).

**Figure 9:**
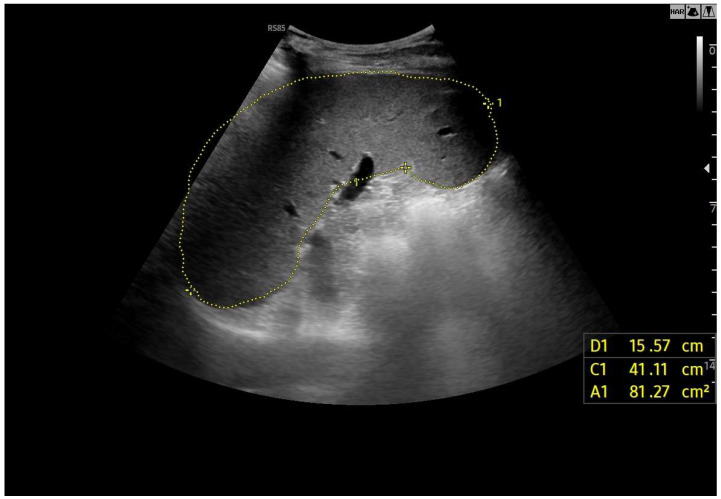
Splenomegaly: bipolar diameter 16 cm (normal value: 12 cm); sectional area 81 cm^2^ (normal value: 45 cm^2^).

A cohort analysis involving 55 individuals who had undergone the Fontan procedure demonstrated that 67% displayed irregular hepatic echotexture or surface nodularity, changes that showed a temporal association with the duration since surgery. Severe congestion with PHTN may present with dilated portal veins (diameter > 13 mm), enlarged paraumbilical veins, collateral circulation, splenomegaly (Figure 9), ascites, and reversal of portal vein flow [50,52,53].

Compared to liver cirrhosis from other causes, extrahepatic portosystemic collaterals in FALD tend to be infrequent or of limited size, even when liver disease becomes decompensated. This occurs because the Fontan circulation generates markedly elevated systemic venous pressures while maintaining only a minimal gradient between the portal and systemic venous systems, conditions that hinder the development of substantial portosystemic collateral pathways [54]. Doppler ultrasonography represents the most informative imaging modality for evaluating liver involvement. It provides detailed information on blood flow and supports the assessment of portal hypertension as well as secondary findings such as splenomegaly, hepatomegaly, ascites, and hepatic nodules [55].

**Figure 10:**
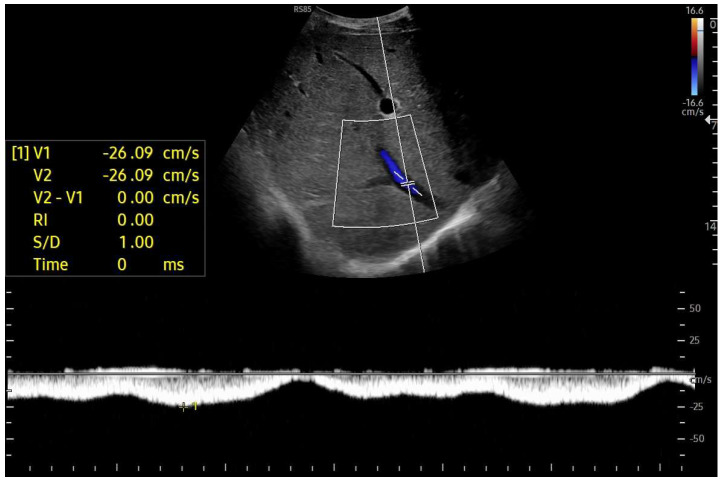

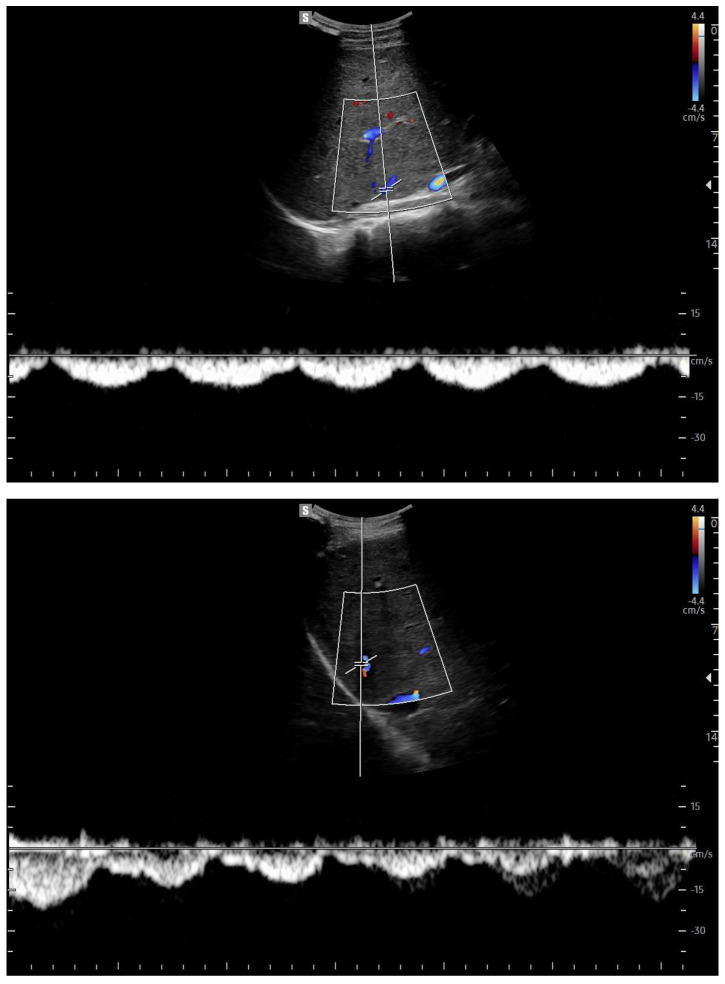
Doppler Ultrasound of the middle hepatic vein showing the loss of the normal three-phase pattern due to the absence of atrial contraction.

**Figure 11:**
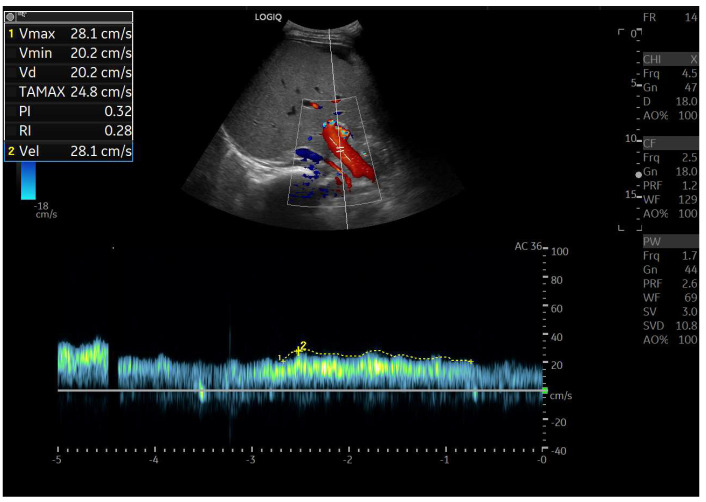
Preserved hepatopetal portal flow in atrio–pulmonary connection.

**Figure 12:**
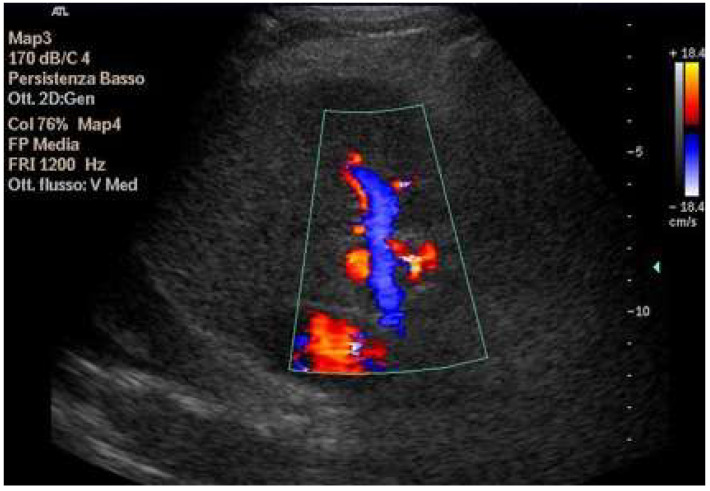
Inverted portal flow in cavo-pulmonary anastomosis.

**Figure 13:**
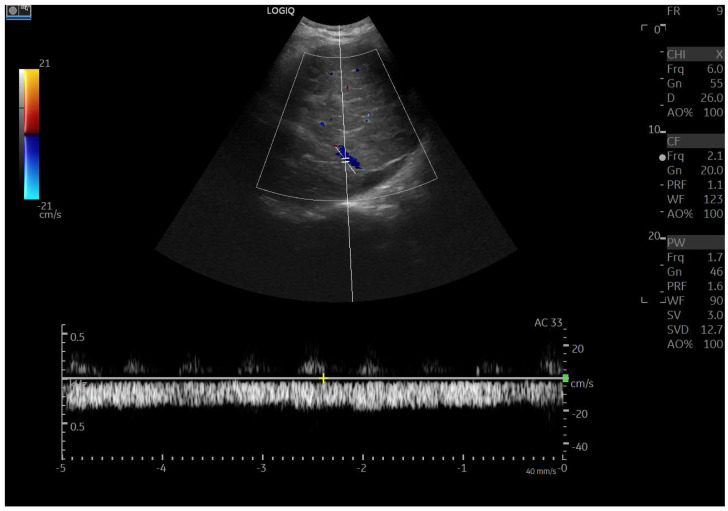
Changes in hepatic vascularization in a patient with FALD. The middle hepatic vein is patent, although irregular, within the context of the parenchymal nodularity.

Doppler patterns in Fontan patients resemble those observed in chronic liver disease, including reduced portal flow velocity (mean flow velocity < 14 cm/s) [56]. The Fontan procedure inevitably alters hepatic venous waveforms on Doppler US (Figure 10, Figure 11, Figure 12 and Figure 13). Inverted portal flow has a specificity of 100% for diagnosing PHTN [57] (Figure 12). The hepatopetal phase pattern in the hepatic vein differs between patients with total cavo-pulmonary anastomosis (including both lateral tunnels and extracardiac conduits) and those with atrio–pulmonary connection [58,59,60]. In atrio–pulmonary connection, hepatopetal flow is preserved (Figure 10), reflecting the exclusion of atrial contribution to venous circulation, whereas in total cavo-pulmonary anastomosis, flow reversal (Figure 11) occurs only during early expiration. Similarly to congestive heart failure, hepatic veins and the IVC are dilated, with abnormally increased hepatic vein pulsatility, regardless of the anastomosis technique [54,61,62]. The loss of the normal three-phase Doppler pattern in hepatic veins is universal following bi-cavo-pulmonary surgery due to the absence of atrial contraction. The presence of a monophasic pattern indicates advanced liver injury [63].

Among individuals with Fontan physiology, the hepatic veins typically show a dampened, predominantly hepatopetal monophasic waveform, reflecting the presence of long-standing hepatic congestion. Venous flow velocities are markedly reduced compared with those measured in healthy individuals [63,64]. In contrast, in later stages characterized by cirrhosis and increased hepatic stiffness, further dampening or paradoxical changes in the venous flow pattern may be observed [37,56,64].

In particular, the hepatic venous Doppler waveform offers valuable insight into hemodynamic changes over time. In the early post-Fontan period, in the absence of atrial contraction and before structural liver damage is evident, the hepatic venous flow may display a relatively blunted monophasic or biphasic waveform with reduced phasicity, reflecting the lack of pulsatility and elevated central venous pressure [37,56,64].

As FALD progresses, and especially in the setting of developing cirrhosis, the Doppler waveform may become more dampened or even flat, corresponding to the increasing stiffness of the hepatic parenchyma, reduced compliance of the vascular bed, and worsening portal hypertension [37,56,64]. Simultaneously, the hepatic veins may appear dilated in the early and mid-stages of disease due to chronic venous congestion, but may show reduced caliber in later stages as fibrosis progresses and vascular remodeling occurs [37,56,64].

Tracking these Doppler changes, from waveform morphology to vein diameter, could provide a non-invasive, dynamic marker of FALD evolution. Including representative Doppler images at different timepoints post-Fontan would help clinicians recognize these transitions and potentially stratify risk or guide surveillance intensity.

Interestingly, hepatic vein waveform analysis can also serve as a surrogate marker for cardiac index, providing indirect insight into the hemodynamic status of Fontan circulation. Post-Fontan, IVC and hepatic vein dilation is common, but the main and intrahepatic portal veins tend to be small, likely due to reduced portal perfusion secondary to increased sinusoidal pressure and venous stasis [65]. The lumen width depends on the severity of the liver cirrhosis and, presumably, the duration of the disease. In FALD, the chronic elevation of central venous pressure leads to venous congestion and dilation of the hepatic veins and IVC due to the following reasons: the Fontan circulation results in chronically elevated systemic venous pressure, which is transmitted directly to the hepatic veins via the IVC [37,56,64]. Over time, this causes passive venous dilatation, which can persist or even worsen despite progression to fibrosis and cirrhosis. Thus, even in advanced congestive cirrhosis from FALD, the hepatic veins and IVC often remain dilated [37,56,64]. This is in contrast to classic cirrhosis from other etiologies, where fibrotic contraction can narrow venous structures.

A controlled study of 106 individuals by Kutty et al. found higher resistance and pulsatility indices in the celiac trunk and mesenteric artery, along with a significant decrease in portal velocity in Fontan patients [64]. These changes can be observed in cirrhosis of any etiology as they reflect hemodynamic consequences of cirrhosis in general. In advanced cirrhosis, increased intrahepatic vascular resistance leads to reduced portal inflow, which causes a compensatory increase in arterial inflow. Over time, changes in downstream compliance and resistance cause elevated RI and PI in splanchnic arteries. In FALD, the portal flow is often lower than in other cirrhosis types, and hepatic artery compensation may be more marked. Tellez et al. [66,67] describe that the portal hypertension model in FALD is characteristically hypodynamic and arterial splanchnic perfusion may also be impaired, as shown in Doppler studies. Indeed, this is what makes FALD a unique entity.

## 8. Contrast-Enhanced Ultrasound (CEUS) in FALD

**Figure 14:**
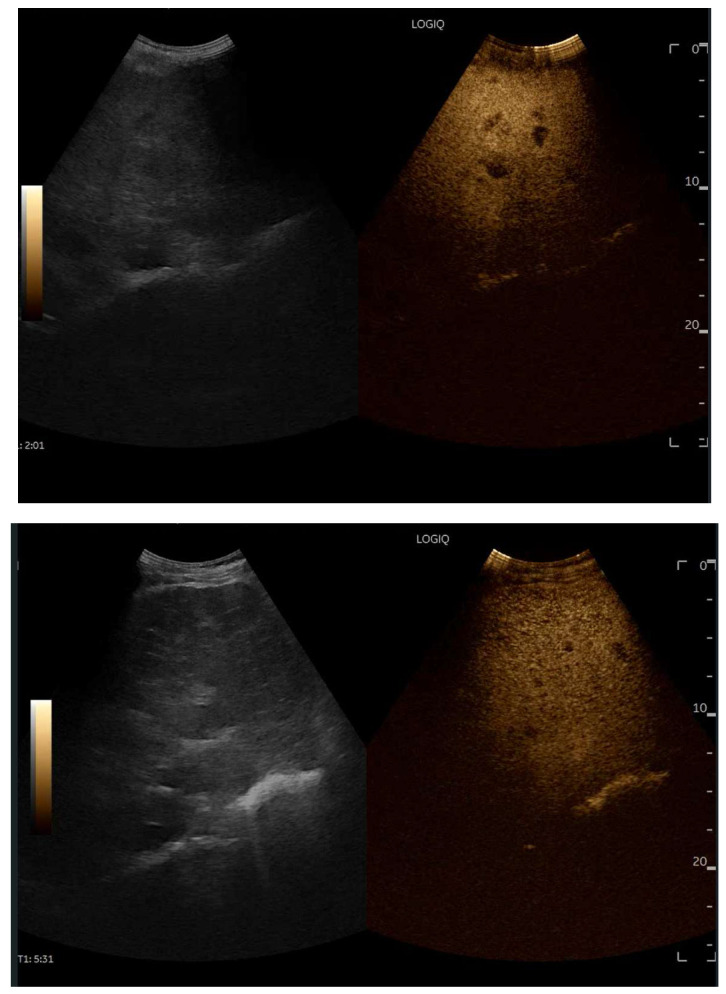
Contrast-enhanced ultrasound (CEUS) in the late phase shows the appearance of hypo-vascular areas due to varying degrees of congestion and hepatic alteration. These areas may mimic malignant nodular lesions with early washout.

**Figure 15:**
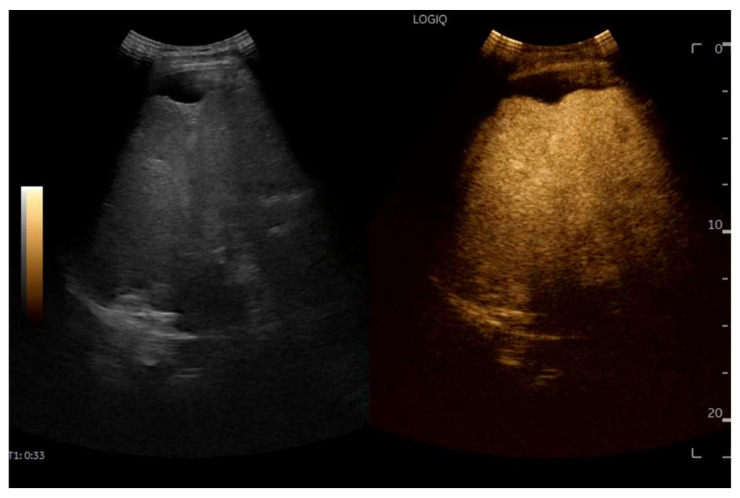
CEUS exam showing late heterogeneous enhancement in the arterial phase with evidence of hypo-enhanced areas, thus mimicking a malignant wash-out.

CEUS is valuable for characterizing the contrast enhancement patterns of liver nodules [68,69,70,71]. In Europe, the US contrast agent SonoVue^®^ (sulfur hexafluoride; Bracco, Milan, Italy) is approved only for intravenous applications in patients over 18 years of age [72]. CEUS use in Fontan patients remains limited due to their frequent collateral circulation with right-to-left shunts, which is considered a contraindication in Europe but not in the United States. Beyond structural liver changes, CEUS reveals markedly heterogeneous hepatic enhancement with mosaic or reticular patterns, mainly due to slow and reduced enhancement near congested hepatic veins—one of the most common imaging features of FALD [54,73]. Anecdotal experience suggests that CEUS demonstrates heterogeneous and decreased liver enhancement in the portal venous phase, similar to cirrhosis of other etiologies (Figure 14, Figure 15). Abnormal enhancement is more prominent at the liver periphery than centrally, while the hypertrophic caudate lobe often shows more homogeneous enhancement. In patients with FALD, the altered hemodynamics resulting from chronic hepatic venous congestion and low cardiac output can significantly influence CEUS dynamics. Despite these circulatory changes, CEUS in FALD generally preserves the standard temporal enhancement pattern. The hepatic veins, although congested, do not typically show early enhancement, as microbubble distribution primarily reflects arterial input. The systemic hypokinetic circulation characteristic of FALD may lead to delayed arterial arrival times and a prolonged transit of contrast, especially in the background of the liver. Nonetheless, FNH-like nodules frequently exhibit intense and early arterial-phase hyperenhancement, suggesting preserved or increased arterial supply in these lesions. This contrast behavior is essential for lesion characterization.

**Figure 16:**
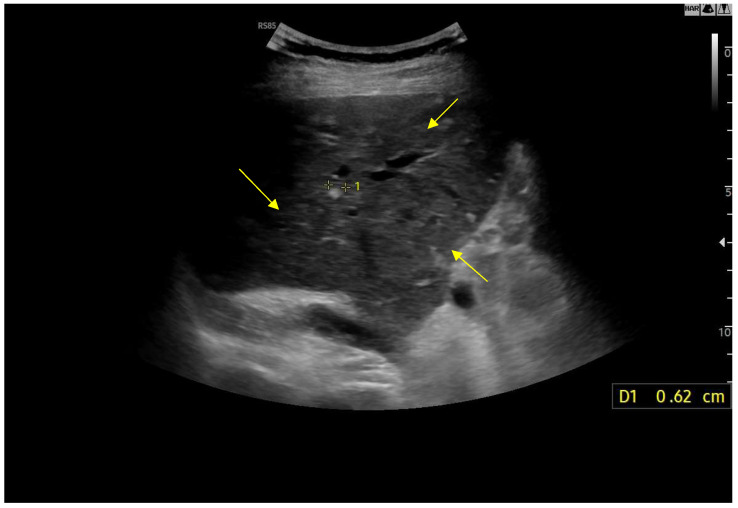
Diffusely dense and heterogeneous echostructure due to the presence of multiple and diffuse hyperechoic areas consistent with FNH-like nodules.

**Figure 17:**
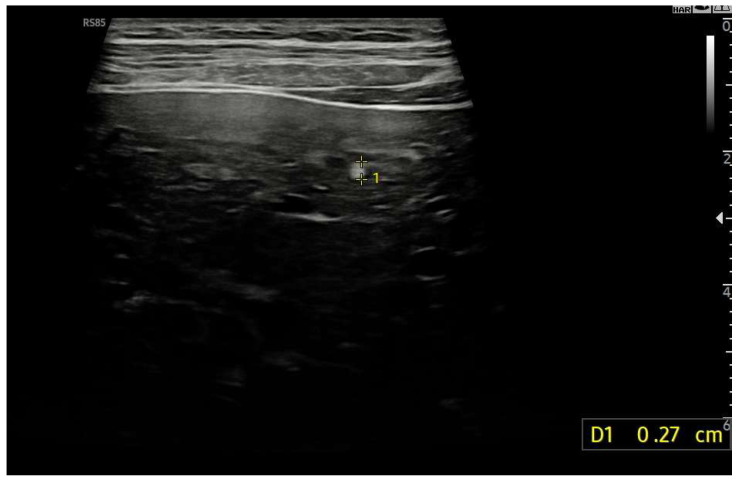
Hyperechoic oval lesion of 2.7 mm, compatible with a FNH-like nodule, identified using a high-frequency linear probe.

CEUS plays a crucial role in evaluating hepatic nodules in FALD and Figure 16 (Figure 17). In non-cardiac cirrhosis, contrast washout in the late phase is highly indicative of HCC. However, in FALD and other congestive hepatopathies like Budd–Chiari syndrome, FNH-like nodules can also exhibit delayed washout, leading to false positives if the LI-RADS system is strictly applied [74]. An FNH-like lesion is a benign hyper-vascular regenerative nodule that mimics focal nodular hyperplasia on imaging but arises secondary to chronic hepatic venous congestion and altered perfusion, typically occurring in FALD or other forms of congestive hepatopathy. Washout in the portal venous phase, in contrast, is uncommon in FNH-like nodules and more specific for HCC [69]. Despite limitations, ancillary LI-RADS criteria and portal venous phase washout remain useful for identifying potentially malignant lesions [75,76].

Therefore, CEUS in FALD must be interpreted with awareness of the altered vascular physiology, and ideally integrated with MR findings and clinical context for accurate diagnosis and longitudinal monitoring.

## 9. Ultrasound Differentiation of Liver Nodules

In FALD patients, these lesions are typically multiple, hyper-vascular, and hyperechoic, generally measuring less than 3 cm and positioned along the peripheral contours of the liver. Hyper-vascular hepatic nodules occur frequently in individuals with Fontan, most of which correspond to benign regenerative lesions or focal nodular hyperplasia (FNH) [66] (Table 6). Although the exact mechanisms driving nodule formation remain uncertain, their subcapsular distribution and imaging features point toward a predominantly vascular pathogenesis. It has been proposed that central venous hypertension—conveyed directly to the perivenular hepatic microcirculation—diminishes portal perfusion and favors progressive arterialization [77]. In adults with Fontan circulation, these nodules are reported in approximately 20% to 30% of cases [66]. These lesions, often termed enlarged regenerative nodules or FNH-like lesions, have also been described in disorders associated with impaired hepatic venous drainage, including Budd–Chiari syndrome and right-sided heart failure [66].

**Figure 18:**
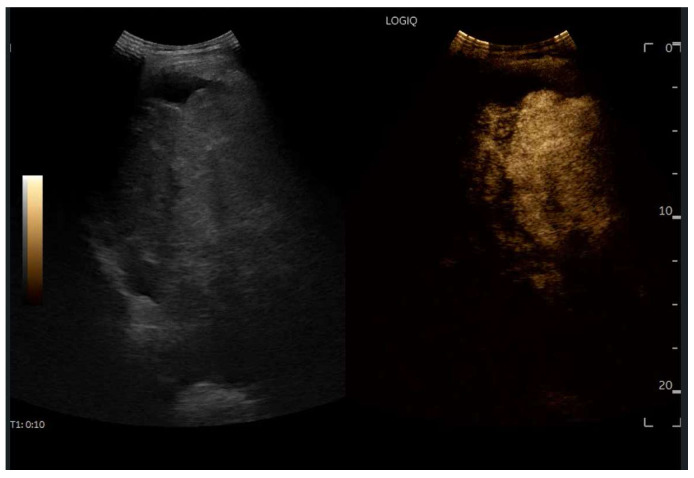
Contrast-enhanced ultrasound (CEUS) in the arterial phase; note the heterogeneous wash-in of multiple hepatic areas.

**Figure 19:**
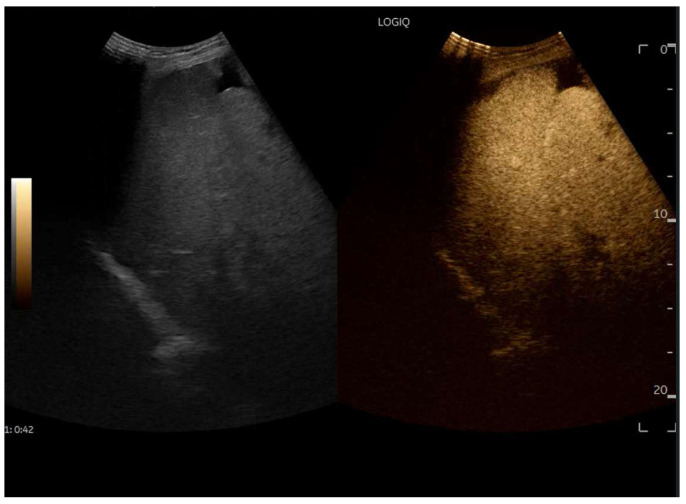
Contrast-enhanced ultrasound (CEUS) in the portal phase; The enhancement of the different hepatic regions becomes homogeneous.

FNH-like nodules in FALD tend to be small, multiple, and predominantly located in the right lobe near the liver surface and within 2 cm (Figure 18 and Figure 19) [78]. On B-mode evaluation, FNH-like nodules often appear as small, hyperechoic, and sometimes multiple lesions, mostly located in the right hepatic lobe [77]. In contrast-enhanced ultrasound (CEUS), FNH-like nodules in FALD exhibit enhancement patterns similar to FNH, including hyperenhancement in the arterial phase, centrifugal enhancement, central stellate vasculature, and sustained enhancement without washout [77]. These nodules may demonstrate central hypo-enhancement in the portal and late phase, corresponding to a central scar [77]. On CT, arterial phase nodules appear hyper-enhancing compared to the surrounding liver parenchyma. In portal and delayed phases, they generally remain iso- or hyperattenuating, but occasional washout may be seen, due to background parenchymal congestion. On MRI T1- and T2-weighted sequences, the nodules are isointense or mildly hypointense on T1 and isointense or mildly hyperintense on T2 [79]. A central scar, hypointense on T1 and hyperintense on T2, may be present [79]. In the hepatobiliary phase, FNH-like nodules retain contrast and appear hyperintense [79]. This helps differentiate them from HCC, which usually appears hypointense in this phase. On DWI (Diffusion-weighted imaging), these nodules do not restrict diffusion, in contrast to HCC, which often shows diffusion restriction [79].

## 10. Hepatocellular Carcinoma (HCC) in FALD

HCC may arise in adult patients with FALD [80]. Routine HCC surveillance in FALD has been recommended, although agreement is lacking regarding optimal intervals, preferred imaging modalities, and the most appropriate timing after the Fontan procedure [3,66]. In Fontan-related cirrhosis, progression from a dysplastic nodule to HCC is thought to occur through sequential pathological steps. Interval size increase, portal-phase washout, mosaic architecture, and elevated alpha-fetoprotein levels can raise concern for malignancy [36,54].

HCC typically presents with arterial-phase hyperenhancement followed by mild late washout on dynamic contrast-enhanced imaging [29,81,82,83,84]. Suspicion for HCC rises in the presence of large lesions, interval enlargement, altered echogenicity, a mass-like configuration, or nodules that distort the liver’s contour. FNH-like nodules often show arterial hyperenhancement and no washout on CEUS, mimicking classic FNH. However, some may exhibit delayed washout, raising suspicion for HCC. Therefore, correlation with MRI and clinical context is essential to avoid misdiagnosis and unnecessary interventions [77,85]. Wells et al. suggested that washout may not indicate a primary abnormality of the nodule, but rather the background hepatic parenchyma retaining contrast due to congestion and fibrosis [86]. Screening every 4–12 months using radiological techniques is considered cost-effective and associated with improved survival in cirrhotic patients. Most experts advocate repeated surveillance, although the most suitable imaging strategy and monitoring interval have yet to be defined [87,88]. HCC treatment should adhere to conventional clinical practice guidelines applied to cirrhosis of other causes [89].

## 11. Liver & Spleen Stiffness

**Figure 20:**
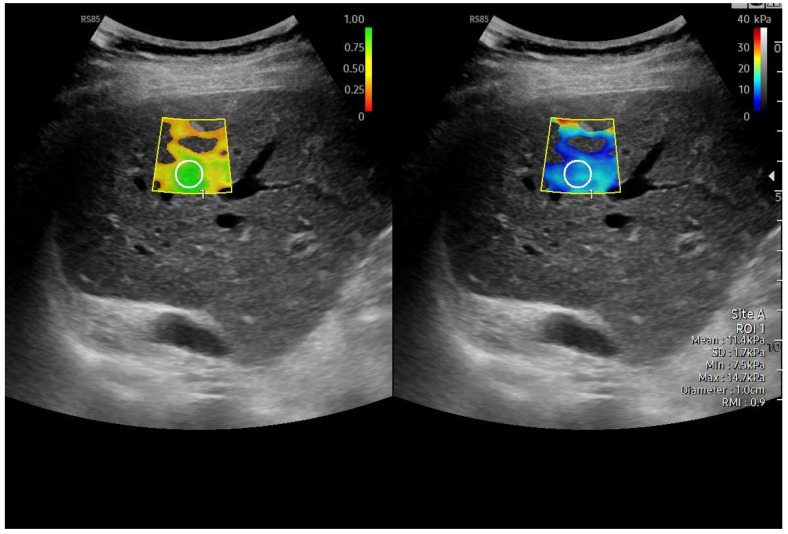
Liver elastography performed using the 2D-SWE method. Left image: reliability color map of the elastography acquisition. The scale ranges from red (low reliability) to green (highest reliability), indicating that the shear-wave propagation in the sampled area was stable and technically adequate. Right image: quantitative stiffness measurement obtained from the region of interest (ROI), showing a mean value of 11.4 kPa with an IQR/M of 9%, consistent with F4 fibrosis in the clinical context of Fontan-associated liver disease.

Different elastography techniques have been investigated for liver stiffness measurement (LSM) in Fontan patients, including point shear-wave elastography (also known as point radiation force impulse), two-dimensional shear-wave elastography imaging, transient elastography (TE) (Fibroscan^®^), and MR elastography (MRE) [90,91,92,93,94,95,96,97]. However, their application in Fontan patients remains challenging because systemic venous congestion can lead to an overestimation of LSM (Table 7). Fontan surgery results in an immediate increase in liver stiffness (LS) due solely to hepatic congestion [98] (Figure 20). Over time, signs of Fontan failure typically emerge, leading to further increases in LS values, which may exceed 15 kPa levels indicative of advanced disease stages in non-congestive liver diseases. In these cases, fibrosis progression may play a role [31,87]. Real-time tissue elastography (RTE) has been shown to reflect liver fibrosis in FALD from the early stages. When combined with hepatic vein waveform analysis, RTE has proven to be a valuable non-invasive tool for evaluating clinical conditions in FALD patients [37].

US elastography, particularly shear-wave techniques, is increasingly being used for serial follow-up in these patients [11,99,100,101,102,103,104,105,106,107,108,109]. Longitudinal LSM assessment during follow-up could help monitor disease progression and predict clinical outcomes. However, elastography results must be interpreted with caution in the Fontan population, as no validated cut-off values for severe liver fibrosis exist for this specific group, unlike in other forms of chronic liver disease [11,99,100,101,102,103,104,105,106,107,108,109,110].

**Table 7:** This table provides a detailed comparative overview of key studies evaluating liver stiffness in Fontan-associated liver disease (FALD). List of Abbreviations: FALD (Fontan-Associated Liver Disease), LSM (Liver Stiffness Measurement), TE (Transient Elastography), SWE (Shear Wave Elastography), 2D SWE (Two-Dimensional Shear Wave Elastography), SWD (Shear Wave Dispersion), MRE (Magnetic Resonance Elastography), MRI (Magnetic Resonance Imaging), CT (Computed Tomography), US (Ultrasound), LFI (Liver Fibrosis Index), APRI (AST to Platelet Ratio Index), FIB-4 (Fibrosis-4 Index), LSPS (Liver Stiffness-Spleen Diameter to Platelet Ratio Score), MELD (Model for End-Stage Liver Disease), AFP (Alpha-Fetoprotein), AUROC (Area Under the Receiver Operating Characteristic Curve), HCC (Hepatocellular Carcinoma).

Study	Sample Size	Modality	Comparator	Main Findings
Sakae et al. (2025) [109]	37	TE (FibroScan^®^)	Serum markers (FIB-4, GGT), clinical parameters	FIB-4, GGT, and age at Fontan were independently associated with elevated LSM; TE correlated with liver fibrosis risk.
Imoto et al. (2025) [108]	46	SWE, Platelet count, LFI	Liver biopsy results	Platelet <185k + LFI> 2.21 predicted aFALD with high sensitivity; proposed a 2-step algorithm for screening.
Lo Yau et al. (2025) [111]	29	US elastography, Liver biopsy	METAVIR vs. congestive hepatic fibrosis score	Weak correlation between US elastography and histologic fibrosis. 86% had METAVIR > F2; median shear wave 1.97 m/s.
Yau et al. (2025) [112]	56	SWE, Hemodynamic assessment	Cardiac catheterization, Echocardiography parameters	No significant correlation between SWE and pre-/post-Fontan hemodynamics. No association with systolic function or AV valve regurgitation on echo. Liver stiffness measurements appear independent of cardiac output parameters.
Cuadros et al. (2025) [107]	91	TE (FibroScan^®^)	Clinical outcomes, fibrosis markers	Elevated LSM predicted major events and mortality in Fontan patients; validated prognostic value of TE.
Téllez et al. (2025) [106]	217	TE, Biopsy, FonLiver score	Other non-invasive tests (APRI, FIB-4, MELD)	FonLiver score outperformed traditional markers; strong diagnostic tool combining TE and platelet count.
Bolia et al. (2024) [105]	48	SWE	Liver stiffness, biomarkers	SWE showed weak correlation with established FALD markers; limited predictive utility.
Jarasvaraparn et al. (2024) [11]	66	APRI, FIB-4, TE	Histologic and imaging findings	APRI and FIB-4 were moderately predictive in adults but not effective in children.
Gill et al. (2023) [104]	25	2D SWE, TE	Liver stiffness measurements	Poor concordance between SWE and TE; questioned reliability of elastography.
Nagasawa et al. (2022) [103]	27	SWD, 2D SWE	Fibrosis stage (biopsy)	SWD more accurate than SWE for detecting significant fibrosis.
Chemello et al. (2021) [102]	52	TE	Portal hypertension and clinical stage	LS and LSPS effectively staged FALD and identified portal hypertension risk.
Schleiger et al. (2020) [101]	145	TE, FibroTest^®^, US	Fontan duration, clinical severity	Fibrosis scores strongly associated with Fontan duration and clinical severity.
Munsterman et al. (2019) [100]	49	US, TE, MRI/CT, Biopsy	Histology and imaging findings	Universal fibrosis found; varices and nodules inconsistently reflected severity.
Egbe et al. (2018) [99]	22	MRE	MELD score, outcomes	Annual MRE-LS progression correlated with worsening MELD and clinical outcomes.

A few small studies have been published correlating spleen stiffness measurement (SSM) with liver fibrosis, but no firm conclusions can be drawn [109,113,114,115]. One prospective study reported that SSM was higher in children with Fontan circulation than in healthy controls [116]. On the other hand, a retrospective study found SWE-SSM to be comparable to that of healthy controls [115]. Thus, more studies are needed to evaluate the role of spleen stiffness measurement in staging FALD.

## 12. Liver Ultrasound-Guided Biopsy

Liver biopsy remains the reference diagnostic method and is still required to accurately assess the degree of hepatic injury [48,117,118]. Typical histological features of FALD include sinusoidal dilatation, perisinusoidal fibrotic changes, centrilobular hemorrhagic necrosis, ductular proliferation, periportal fibrous septation, central fibrous bridging, and nodular regenerative hyperplasia. Sinusoidal dilation is present in 90–97% of Fontan patients and is the earliest parenchymal change. Typically, it is more pronounced than in other causes of cardiogenic hepatopathy. The distribution of fibrosis in the early stages is typically perisinusoidal (in the space of Disse), a pattern that distinguishes it from other types of heart-related liver injury. In more advanced stages, broad bands of centrilobular fibrosis accompanied by regenerative nodules typically emerge. Periportal inflammatory activity is often scant or entirely lacking, a feature that assists in distinguishing FALD from alternative hepatic conditions [119]. Certain experts advocate performing a liver biopsy routinely at approximately 10 years post-Fontan procedure [87]. In a cohort of 67 patients, this strategy revealed that all patients developed hepatic fibrosis, and its extent increased over time. However, the authors found no correlation between the degree of fibrosis and clinically relevant events, hemodynamic, or analytical parameters [120], casting doubt on its practical usefulness in routine clinical management. At present, liver biopsy is advised when the cause of hepatic disease remains uncertain or when evaluating candidates for heart and/or liver transplantation [64,121,122].

## 13. FALD Follow-Up

Both the 2019 American Heart Association (AHA) scientific statement and the 2023 EASL-ERN position paper emphasize that FALD requires lifelong, structured, and multidisciplinary surveillance conducted in specialized Fontan clinics [3,123,124,125]. (Table 8). Although high-quality evidence remains limited, there is a broad consensus that follow-up must integrate cardiac, hepatic, and end-organ assessment across childhood and adulthood. Cardiac surveillance should include regular cross-sectional imaging—preferably cardiac MRI every 2–3 years—to evaluate ventricular function, Fontan pathway anatomy, and flow dynamics [3,123].

In patients with contraindications to MRI, CT could be performed to evaluate Fontan anatomy and various postoperative complications; however, when detailed hemodynamic data are required, cardiac catheterization is considered appropriate [126].

Hepatic surveillance relies on annual liver ultrasound with elastography as the first-line modality, with shorter intervals when signs of fibrosis or nodularity are present [3]. Both societies recommend obtaining a baseline liver MRI with hepatobiliary contrast at ~10 years post-Fontan, followed by MRI every 1–2 years, particularly for high-risk individuals [3,45,125,127,128]. MRI is preferred to CT for lesion characterization, though its use may be limited in patients with legacy devices [125,127]. Because classical LI-RADS criteria are less reliable in congestive hepatopathy, biopsy remains essential when imaging findings are indeterminate [5].

Given the rising incidence of HCC—estimated between 0.6% and 4.9% within two decades post-Fontan—both guidelines endorse active HCC surveillance starting at least 10 years after Fontan completion, or earlier in patients with Fontan circulatory failure, elevated non-invasive fibrosis scores (FIB-4, APRI, MELD > 19) [129], or other high-risk features. Semiannual ultrasound combined with periodic contrast-enhanced imaging offers a practical approach for detecting and characterizing nodules [3,78,129,130].

Serum AFP serves as a useful adjunct biomarker for identifying HCC in FALD, as 74–80% of affected patients exhibit values exceeding standard upper limits [128]. Serial AFP measurement has been suggested to be incorporated into routine follow-up. An AFP value above 10 ng/mL has been identified as a strong predictor of HCC, and should prompt more aggressive diagnostic work-up, including advanced imaging or biopsy [3]. In a large prospective study with a high rate of non-neoplastic nodules (48%), none of the Fontan patients without HCC had AFP levels above 7 ng/dL [45]. In a sizable cohort of stable Fontan patients, AFP concentrations above 10 ng/dL were associated with a 26-fold higher risk of HCC [42]. Notably, individuals who did not develop HCC demonstrated considerably lower AFP values (median 2.9 ng/dL), reinforcing its predictive value for new-onset HCC in this population [42]. Accordingly, elevated AFP levels in FALD should consistently prompt clinical suspicion for HCC. Therefore, surveillance intervals should remain flexible, with the frequency of imaging and biomarker assessment tailored to the patient’s evolving risk profile and clinical findings.

**Figure 21:**
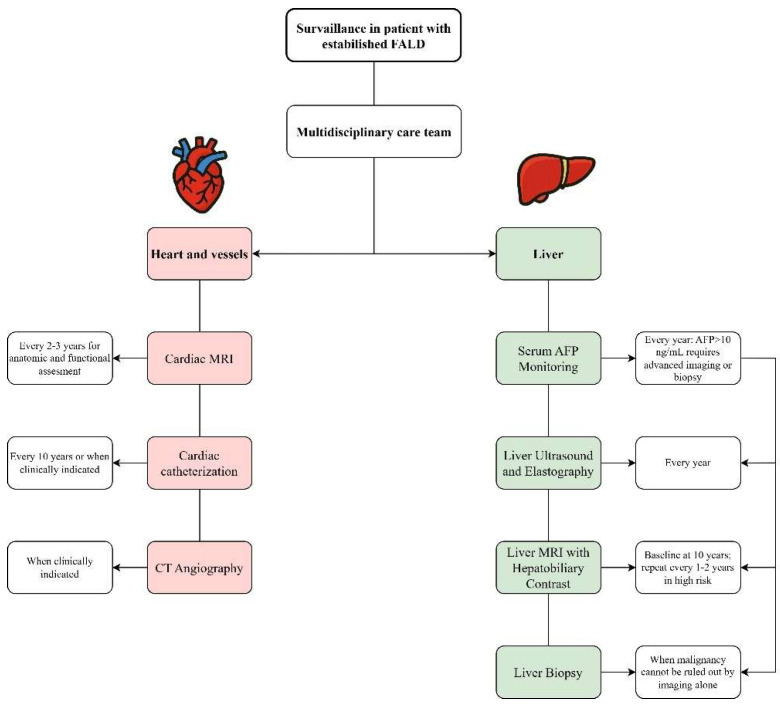
Proposed surveillance algorithm for patients with established Fontan-associated liver disease (FALD). The multidisciplinary care pathway includes parallel assessment of the cardiovascular system and the liver.

A relevant consideration is the need for specialized management of FALD involving both hepatologists and cardiologists with direct expertise in this condition (Figure 21). In clinical practice, many patients are incorrectly labelled as having “cirrhosis” solely based on elevated liver stiffness values—often obtained through transient elastography—without adequate contextual interpretation. Because liver stiffness in Fontan patients is strongly influenced by venous congestion and architectural distortion rather than classical fibrotic cirrhosis, misclassification is common. This can lead to unnecessary diagnostic procedures, inappropriate surveillance pathways, and significant patient anxiety. Given these complexities, a centralized, multidisciplinary care model is likely to provide the most accurate assessment and management. Teams experienced in FALD can integrate haemodynamic findings, cardiac physiology, imaging, elastography, and laboratory parameters to distinguish congestion-related changes from true advanced fibrosis. Such an approach not only may improve diagnostic accuracy but also ensures that surveillance, risk stratification, and therapeutic decisions are evidence-based and tailored to the unique pathophysiology of the Fontan circulation.

## 14. Future Directions

Several unanswered questions remain in the assessment and management of FALD. A central challenge is distinguishing true hepatic fibrosis from changes driven predominantly by chronic cardiac congestion. Elevated liver stiffness, nodular morphology, and biochemical abnormalities can all reflect haemodynamic derangements rather than irreversible structural liver disease, making accurate diagnosis difficult. Future work should prioritize approaches that integrate cardiac and hepatic parameters rather than relying on isolated measurements. Tools such as the recently proposed FonLiver score, which adjusts liver stiffness for thrombocytopaenia to better identify cirrhosis, represent an important step toward combined assessment [106]. However, thrombocytopaenia itself is influenced by both hepatic dysfunction and elevated venous pressures in decompensated Fontan circulation, underscoring the need for more refined models [12]. Incorporating direct or surrogate measures of haemodynamics—such as Fontan pressure, systemic venous congestion indices, or MRI-based flow and stiffness mapping—may provide a more granular and physiologically grounded evaluation. Future studies should therefore aim to validate multimodal, longitudinal diagnostic frameworks that integrate elastography, laboratory markers, detailed imaging, and haemodynamic assessment. Such an approach is likely to improve diagnostic precision, guide risk stratification, and ultimately enhance clinical decision-making regarding surveillance, timing of intervention, and consideration for heart or combined heart–liver transplantation.

## 15. Conclusions

Although the Fontan procedure has extended patients’ lifespan, there remains a significant risk of morbidity and mortality. Patients undergoing the Fontan procedure at birth require lifelong follow-up with medical care, which has a considerable financial and societal impact. However, much remains unknown about how Fontan palliation affects patients differently. End-organ effects are increasingly recognized, with particular attention to cardiac, lymphatic, and liver changes, which require monitoring through radiological techniques to ensure optimal timing for treatment and, eventually, cardiac transplantation or combined liver–heart transplantation. FALD is an emerging liver disease that poses a significant clinical challenge. A FALD surveillance program should be recommended to detect liver disease progression at an early stage, allowing for interventions that optimize Fontan circulation and prevent advanced liver fibrosis. Multi-institutional collaborative registries and prospective studies are essential.

## Figures and Tables

**Table 1:** Clinical and hemodynamic phenotypes of Fontan Circulation Failure.

Phenotype	Ventricular Function	Fontan Pressure	Cardiac Index	Key Clinical Features	Prognosis
Systolic Fontan Phenotype	Reduced	Variable	Low	Heart failure symptoms	Poor, high transplant risk
Preserved Fontan Phenotype	Normal	Elevated	Normal or mildly low	Systemic congestion	Intermediate
Lymphatic Fontan Phenotype	Variable	Normal or low	Variable	Protein-losing enteropathy, plastic bronchitis	High morbidity
Normal Hemodynamics Phenotype	Normal	Normal	Normal	Nonspecific symptoms	Variable, challenging diagnosis

**Table 2:** Similarities between FALD and non-cardiac cirrhosis. HCC, hepatocellular carcinoma; FALD, Fonta-associated liver disease. MASH, metabolic dysfunction-associated steatohepatitis.

FEATURE	FALD	VIRAL/AUTOIMMUNE CIRRHOSIS (PORTAL-BASED)	ALCOHOL-RELATED CIRRHOSIS	MASLD/MASH-RELATED CIRRHOSIS
**PRIMARY DRIVER**	Chronic hepatic venous congestion due to elevated CVP	Chronic inflammation starting in portal tracts	Toxic/metabolic injury from alcohol; oxidative stress	Lipotoxicity, metabolic inflammation
**INITIAL FIBROSIS PATTERN**	Predominantly centrilobular/perisinusoidal	Periportal → bridging → cirrhosis	Initially zone 3 (centrilobular) fibrosis	Perisinusoidal (especially zone 3)
**DISTRIBUTION OF FIBROSIS**	Patchy, heterogeneous; may spare portal tracts	Portal-based bands with nodular transformation	Centrilobular early; later bridging & micronodules	Variable; diffuse in advanced disease
**LIVER SIZE**	Early enlargement → later right-lobe atrophy	Often small, shrunken	Often shrunken, nodular	Often enlarged early; may shrink late
**PORTAL HYPERTENSION**	Late, often partly reversible with hemodynamic optimization	Early and progressive	Common in advanced disease	Present and progressive

**Table 3:** Comparison of key clinical, biochemical, histological, and hemodynamic features across Fontan-associated liver disease (FALD) and other etiologies of cirrhosis (e.g., viral, metabolic). CVP = central venous pressure; MASLD = metabolic dysfunction-associated steatotic liver disease; AST = aspartate aminotransferase; ALT = alanine aminotransferase; INR = international normalized ratio.

FEATURE	FALD	VIRAL/AUTOIMMUNE	ALCOHOL-RELATED	MASLD/MASH
**HEMODYNAMICS**	Elevated CVP; low cardiac output	Normal CVP; sinusoidal resistance-driven	Normal CVP	Normal CVP
**TYPICAL AGE AT ONSET**	Childhood/adolescence	Adulthood	Adulthood	Adulthood
**ENZYMES**	Mild AST/ALT ↑; cholestasis variable	Higher AST/ALT; inflammation	AST>ALT typical	Mild ALT>AST
**SYNTHETIC FUNCTION**	Often preserved until late	Declines with disease progression	Variable; may decline early	Usually preserved until advanced
**PLATELETS**	Variable; may be normal early	Often reduced	Often reduced	Reduced with portal hypertension
**COURSE**	Heterogeneous; tied to Fontan hemodynamics	Progressive without treatment	Progressive with heavy use	Slow–mod/severe progression

**Table 4:** The main proposed mechanisms influencing FALD’s development.

POTENTIAL CELLULAR FACTORS	PERIOPERATIVE/PRENATAL FACTORS	POSTOPERATIVE HEMODYNAMIC VASCULAR FACTORS	ADDITIONAL FACTORS
ACTIVATION OF MECHANOSENSITIVE RECEPTORS	-Systemic hypotension	Increased CVP (non-pulsatile)	-Hepatotoxins
-LIVER SINUSOIDAL ENDOTHELIAL CELLS (LSEC)	-Increased CVP	-Congestion	-Alcohol, obesity
-HEPATIC STELLATE CELLS (HSC)	-Hypoxia	-Portal hypertension	-Virus
		-Decreased arterial flow	-Metabolic factors
CYTOKINE ACTIVATION			
-CXCL1, IL6, TNF		Decreased cardiac output	
		-Hypoxia	
MICROVASCULAR THROMBOSIS			
		Increased lymphatic angiogenesis	
NEUROHORMONAL ACTIVATION			
-RENIN, ANGIOTENSIN, ALDOSTERONE		Visceral congestion-ischemia	
		-Intestinal microbiome	
		Abnormal hepatic venous circulation	
		-Venovenous shunts	

**Table 5:** Laboratory scoring systems. ALT, alanine transaminase; AST, aspartate aminotransferase; APRI, aspartate aminotransferase to platelet ratio index; FIB-4, fibrosis-4; MELD, model for end-stage liver disease; MELD-XI, model for end-stage liver disease excluding the international normalized ratio; FALD, Fontan-associated liver disease; INR, International Normalized Ratio; GGT, gamma-glutamyl transferase.

SCORING SYSTEM	VARIABLES	PREDICTION OF FIBROSIS IN FALD
**AST/ALT RATIO**	AST, ALT.	Difficult to evaluate early fibrosis since a mild increase in these serum markers is common due to congestion.
**APRI**	AST, platelet count.	Dedicated to scoring hepatitis C, not validated in FALD. Decrease in platelet count is associated with advanced disease.
**FIB-4**	AST, ALT, platelet count, age.	Performs less well for FALD patients since the majority are < 40 years.
**MELD**	Bilirubin, creatinine, INR, sodium.	Not appropriate in FALD patients with anticoagulant therapy.
**MELD-XI**	Bilirubin, creatinine.	Correlates to biopsy-proven FALD fibrosis. Potential role in predicting outcome for transplantation heart vs. heart– liver.
**FORNS INDEX**	GGT, cholesterol, platelet count, age.	Takes into account GGT which is also commonly increased in congestive hepatopathy.
**POHL SCORE**	AST, ALT, platelet count.	Dedicated to scoring hepatitis C, not validated in FALD. Decrease in platelet count is associated with advanced disease.
**CIRRHOSIS DISCRIMINANT SCORE**	GGT, AST, ALT, INR, upper limit of AST, platelet count, age.	Dedicated to scoring for hepatitis C, not validated in FALD.

**Table 6:** This table summarizes the distinguishing imaging characteristics of FNH-like nodules observed in Fontan-associated liver disease (FALD) compared with typical findings in hepatocellular carcinoma (HCC). Modalities include standard ultrasound (B-mode), contrast-enhanced ultrasound (CEUS), computed tomography (CT), and magnetic resonance imaging (MRI).

Imaging	Imaging Modality	FNH-Like Nodules Features	Differential Clues from HCC
**Ultrasound**	B-mode	Small, hyperechoic, sometimes multiple lesions	HCC may be iso- or hypoechoic, larger, with irregular margins
	CEUS (Contrast-Enhanced Ultrasound)	Hyperenhancement in arterial phase; centrifugal enhancement; central stellate vasculature; no washout	HCC often shows rapid washout in portal/late phase and lacks central stellate pattern
**CT**	Arterial Phase	Hyper-enhancing compared to liver parenchyma	HCC may also be hyper-enhancing but more likely to show irregular margins or washout later
	Portal/Delayed Phase	Generally, iso- or hyperattenuating; occasional mild washout due to parenchymal congestion	HCC often shows true washout, distinguishing it from regenerative nodules
**MRI**	T1-weighted	Iso- or mildly hypointense; central scar hypointense	HCC is often more heterogeneous and may have intense arterial enhancement
	T2-weighted	Iso- or mildly hyperintense; central scar hyperintense	HCC may be more heterogeneous, with areas of necrosis or hemorrhage
	Hepatobiliary Phase	Hyperintense due to hepatobiliary contrast retention (differentiates from HCC)	HCC typically appears hypointense due to lack of contrast uptake
	DWI	No diffusion restriction (helps distinguish from HCC)	HCC typically restricts diffusion (appears hyperintense on DWI)

**Table 8:** This comparative table outlines the practical follow-up and surveillance strategies recommended by the American Heart Association (AHA, 2019) and the European Association for the Study of the Liver/European Reference Network (EASL-ERN, 2023) for patients with Fontan circulation. It includes guidance on multidisciplinary care, imaging modalities, timing of surveillance, and use of biomarkers to monitor Fontan-associated liver disease (FALD) and hepatocellular carcinoma (HCC).

Surveillance Component	AHA Recommendations (2019)	EASL-ERN Recommendations (2023)
Multidisciplinary Care Team	Fontan/single-ventricle clinics with experienced personnel	Specialized clinics with hepatologist, cardiologist, and radiologist
Cardiac MRI	Every 2–3 years for anatomic and functional assessment	Every 2–3 years to evaluate Fontan flow and anatomy
CT Angiography	When clinically indicated	Not specifically emphasized unless MRI is contraindicated
Cardiac Catheterization	Every 10 years or when clinically indicated	When MRI contraindicated or invasive data needed
Liver Ultrasound + Elastography	Annual ultrasound with elastography	Annual surveillance as first-line imaging tool
Liver MRI with Hepatobiliary Contrast	Annual MRI from adolescence; use hepatobiliary contrast if biopsy is considered	Baseline at 10 years; repeat every 1–2 years in high-risk
Liver Biopsy	If imaging is inconclusive and LI-RADS fails to differentiate	Mandatory when malignancy cannot be ruled out by imaging alone
Serum AFP Monitoring	Use AFP > 7 ng/dL as alert threshold; >10 ng/dL = high risk	AFP > 10 ng/mL requires advanced imaging or biopsy
Timing of HCC Surveillance	Start 10 years after Fontan; consider earlier in high-risk patients	Begin at 10 years post-Fontan; earlier if MELD > 19, APRI/FIB-4 elevated

## Data Availability

Not applicable.
